# Proposed multidimensional framework for understanding Chagas disease healthcare barriers in the United States

**DOI:** 10.1371/journal.pntd.0007447

**Published:** 2019-09-26

**Authors:** Colin Forsyth, Sheba Meymandi, Ilan Moss, Jason Cone, Rachel Cohen, Carolina Batista

**Affiliations:** 1 Drugs for Neglected Diseases *initiative*, North America, New York, New York, United States of America; 2 Center of Excellence for Chagas Disease at Olive View-UCLA Medical Center, Sylmar, California, United States of America; 3 Médecins sans Frontières/Doctors Without Borders USA, New York, New York, United States of America; 4 Drugs for Neglected Diseases *initiative*, Latin America, Rio de Janeiro, Brazil; Instituto de Investigaciones Biotecnológicas, ARGENTINA

## Abstract

**Background:**

Chagas disease (CD) affects over 300,000 people in the United States, but fewer than 1% have been diagnosed and less than 0.3% have received etiological treatment. This is a significant public health concern because untreated CD can produce fatal complications. What factors prevent people with CD from accessing diagnosis and treatment in a nation with one of the world’s most advanced healthcare systems?

**Methodology/Principal findings:**

This analysis of barriers to diagnosis and treatment of CD in the US reflects the opinions of the authors more than a comprehensive discussion of all the available evidence. To enrich our description of barriers, we have conducted an exploratory literature review and cited the experience of the main US clinic providing treatment for CD. We list 34 barriers, which we group into four overlapping dimensions: systemic, comprising gaps in the public health system; structural, originating from political and economic inequalities; clinical, including toxicity of medications and diagnostic challenges; and psychosocial, encompassing fears and stigma.

**Conclusions:**

We propose this multidimensional framework both to explain the persistently low numbers of people with CD who are tested and treated and as a potential basis for organizing a public health response, but we encourage others to improve on our approach or develop alternative frameworks. We further argue that expanding access to diagnosis and treatment of CD in the US means asserting the rights of vulnerable populations to obtain timely, quality healthcare.

## Background

According to estimates, over 6.1 million people worldwide are infected with *Trypanosoma cruzi*, the protozoan that causes Chagas disease (CD), and 1.2 million suffer from cardiomyopathy due to advancement of the disease [1–3]. CD causes over 7,500 deaths per year globally and creates a greater burden of mortality and disability-adjusted life years (DALYs) than any other parasitic disease in the Americas [4]. Timely therapy with antitrypanosomal drugs can delay or prevent life-threatening complications from chronic CD, but globally, access to diagnosis and treatment is extremely limited.

The US has the sixth largest national burden of CD in the world, with estimates indicating >300,000 people infected and >30,000 suffering from CD-related cardiomyopathy [2, 5]. Nationally, the economic toll of CD has been estimated at US$129.3 million in healthcare costs (adjusted to 2019 dollars) and 27,590 DALYs annually [6]. The US has a distinct epidemiological scenario. While 326,000–347,000 people with CD living in the US acquired the disease in Latin America, an additional undetermined number were infected within the US. The insects that transmit CD—known as triatomines, kissing bugs, or, in Mexico and Central America, chinches besucones—are found in 27 states across the southern US as part of the natural environment [7]. Several species of mammals, including raccoons, dogs, armadillos, and opossums, are reservoirs for CD [8–11], and autochthonous transmission has been increasingly documented since the US began screening blood donations for CD in 2007 [12].

CD is usually transmitted by triatomines, but congenital transmission, oral ingestion, blood transfusion, and organ transplantation represent additional infection routes [13]. Initial infection is followed by an acute phase, which is usually asymptomatic, yet can be severe or even fatal. CD then enters a long, indeterminate, asymptomatic chronic phase. However, 30%–40% of those infected progress within 10–30 years to an advanced chronic phase that usually involves cardiac complications, including heart failure, hypertrophy, thromboembolism, and sudden death [13, 14]. Others suffer from gastrointestinal and/or neurological complications, sometimes in conjunction with cardiac symptoms [15–17].

Early diagnosis and antiparasitic treatment can prevent or slow the progression of heart failure and other complications of chronic CD [18–20], significantly reducing the burden of premature mortality and morbidity. Moreover, treatment of *T*. *cruzi*–positive women of childbearing age prevents congenital transmission [21, 22]. However, treatment is less beneficial once CD progresses to the advanced chronic phase with cardiac complications [23]. This makes it essential to screen at-risk populations and detect CD early so that timely treatment can be provided.

Despite this urgency, worldwide, only a miniscule fraction of people with CD receive antiparasitic treatment [3, 24, 25]. This also holds true within the US, where 2,407 people with *T*. *cruzi* infection were identified through blood screening from 2007 to 2018 [26]. During this period, the two drugs for CD (benznidazole and nifurtimox) were not Food and Drug Administration (FDA) approved and were only available in the US via Investigational New Drug (IND) Protocols through Centers for Disease Control (CDC), although benznidazole became commercially available in May 2018. From 2007 to 2013, CDC released 422 courses of benznidazole or nifurtimox (about 60 people annually) [27], and from October 2011 until May 2018, 365 patients obtained benznidazole via the IND (about 55 people annually) [28]. CDC provided these drugs as well as assistance with diagnosis free of charge. Relative to estimates of CD prevalence within the US, these numbers mean that <1% of estimated domestic cases were detected and <0.3% received etiological treatment.

Outside of blood and organ donations (the FDA recommends all blood donors are tested once; organ donations are subject to risk-based screening [29, 30]), there is virtually no systematic screening for CD, even in obstetric care settings. Access to diagnosis is constrained by limited test availability, lack of clear guidelines, and low awareness of CD [27]. Furthermore, based on studies among heart disease patients in Los Angeles and New York, there may be a significant burden of CD-related cardiomyopathy that goes undetected in US hospitals [31–33].

What factors explain this stark, pervasive neglect? In this article, we assess barriers to CD diagnosis and treatment in the US.

## Methods

We define a barrier as any factor that either limits the availability of or prevents patients from accessing diagnosis, treatment, and/or clinical management of CD. We use two sources to develop a list of barriers impacting access to diagnosis and treatment for CD in the US: (1) a review of the literature on CD healthcare access and (2) the experience of one of the few US clinics routinely treating CD patients. Although these sources serve as the starting point and principal basis for our description of barriers, we also draw on a broad range of medical, anthropological, and related public health research to present a comprehensive overview of the access landscape, and to provide context and describe each barrier in detail. Although our intention was to list access barriers for CD, we do not assess the relative weight or importance of each barrier, which could vary considerably depending on state-level and local contexts. Also, a single barrier may impact diagnosis, antiparasitic treatment, and management of complications from CD in different ways that we do not fully describe here.

### Literature search

**Fig 1:**
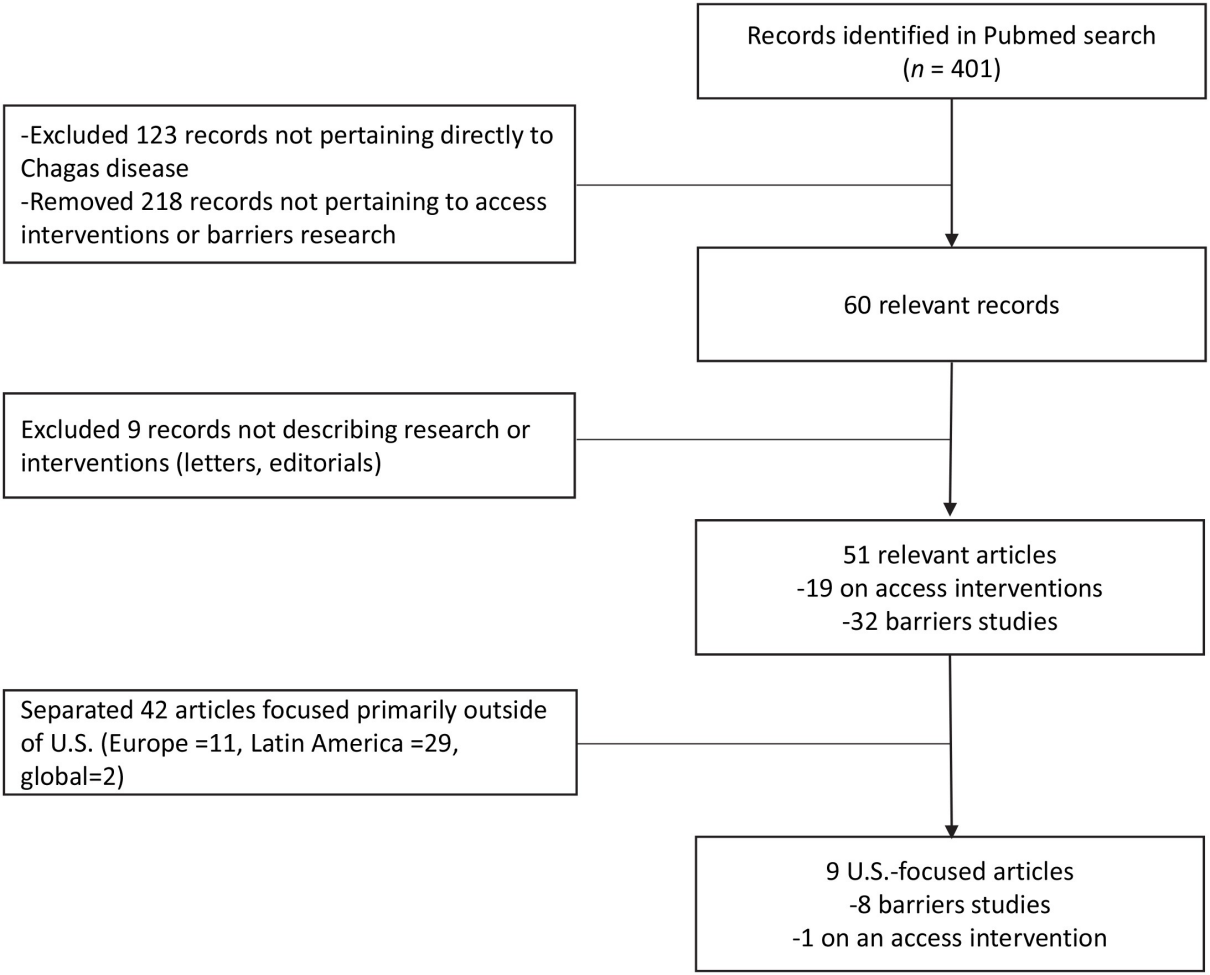
Search strategy for research articles on US access to treatment for CD. CD, Chagas disease.

An exploratory literature search was conducted via PubMed using the keywords “Chagas disease” plus “barriers,” “healthcare,” and/or “access” to identify articles in any language on access to CD healthcare in the US published between January 1, 1980, and July 31, 2018, (Fig 1). Each record’s title and, if necessary, abstract, were reviewed, and records not pertaining to healthcare barriers or access for CD were excluded. Only articles describing original research, systematic reviews, or access interventions were included. Of 51 relevant articles, 19 described access interventions, and 32 represented research on barriers to healthcare. After setting aside articles focusing on Latin America (*n* = 29), Europe (*n* = 11), and global issues (*n* = 2), only 9 articles focused primarily on the US; 1 describes an access initiative and 8 pertain to healthcare barriers (Table 1). However, because these articles do not cover the entire range of access challenges relevant to the US, we also refer to the 42 non-US studies we identified in order to fill in gaps and more fully describe some of the pertinent barriers, particularly in the psychosocial dimension. An important limitation of this approach is that there are substantial differences between the health systems of these countries and that of the US. Furthermore, the US population with CD is heterogeneous, reflecting diverse countries of origin, and sociocultural barriers relevant to people in Argentina, for example, may not be applicable to Mexican-born individuals living in the US. Issues around access and training of health professionals may differ substantially between European or Latin American countries and the US. Moreover, this was an exploratory rather than comprehensive literature search, which is subject to interpretation bias and does not cover a range of studies dealing with the epidemiology, clinical, or other aspects of CD, which, though excluded by the search criteria because their main focus is not access related, still provided important contextual information and are referred to throughout the article.

**Table 1:** Published research on barriers/access to treatment for CD in the US.

Study	Barrier Dimension	Topic
**Stimpert and Montgomery 2010**	Systemic	Survey of US physician knowledge of CD
**Verani and colleagues 2010**	Systemic	Survey of US obstetricians’ knowledge of CD
**Minneman and colleagues 2012**	Systemic	Knowledge of CD among Latin American immigrants in Georgia
**Sanchez and colleagues 2014**	Systemic	Survey of knowledge of CD among Latin American immigrants in Los Angeles
**Manne and colleagues 2015**	Systemic	Health systems analysis of barriers to accessing CD diagnosis and treatment
**Amstutz-Szalay 2016**	Systemic	Knowledge of CD among physicians in Ohio
**Meymandi and colleagues 2017**	Systemic	Description of implementation of a community-based screening program in Los Angeles
**Edwards and colleagues 2018**	Systemic	Survey of Pediatric Infectious Disease Society on CD knowledge
**Forsyth and colleagues 2018**	Multiple	Los Angeles patient perspectives on access to CD treatment
**Additional articles from CECD research**		
**Miller and colleagues 2015**	Clinical	Side effects from benznidazole treatment in patients at the CECD, Los Angeles
**Forsyth and colleagues 2016**	Clinical	Side effects from nifurtimox treatment in patients at the CECD, Los Angeles

CD, Chagas disease; CECD, Center of Excellence for Chagas Disease at Olive View-University of California-Los Angeles Medical Center.

### Insights from a US center of Excellence

The Center of Excellence for Chagas Disease at Olive View-UCLA Medical Center (CECD) is one of the only US providers fully dedicated to comprehensive healthcare for CD, including community-based screening and education, etiological treatment, and management of CD cardiomyopathy. The CECD has screened >8,000 patients for CD and treated >300 since 2007 [34]. When educating, testing, treating, and monitoring patients, and through its efforts to expand the services available to people with CD in Los Angeles and beyond, the CECD has had to contend, directly or indirectly, with the barriers described in this article. We wanted to highlight the lessons learned by the CECD as a basis for better understanding barriers confronting people with CD so that an appropriate public health strategy can be developed. Although some articles from the literature review describe CECD research [34–36], our analysis is meant to provide a more comprehensive and extensive picture that incorporates observations and insights from the CECD’s ongoing activities, which extend far beyond its published research. Additionally, we added two articles to Table 1 (not captured by our literature search) that describe CECD studies of adverse effects from antitrypanosomal drugs [37, 38], bringing the total number of US studies we identified related to diagnosis and treatment barriers to 11.

## A multidimensional framework for understanding CD healthcare barriers

**Fig 2:**
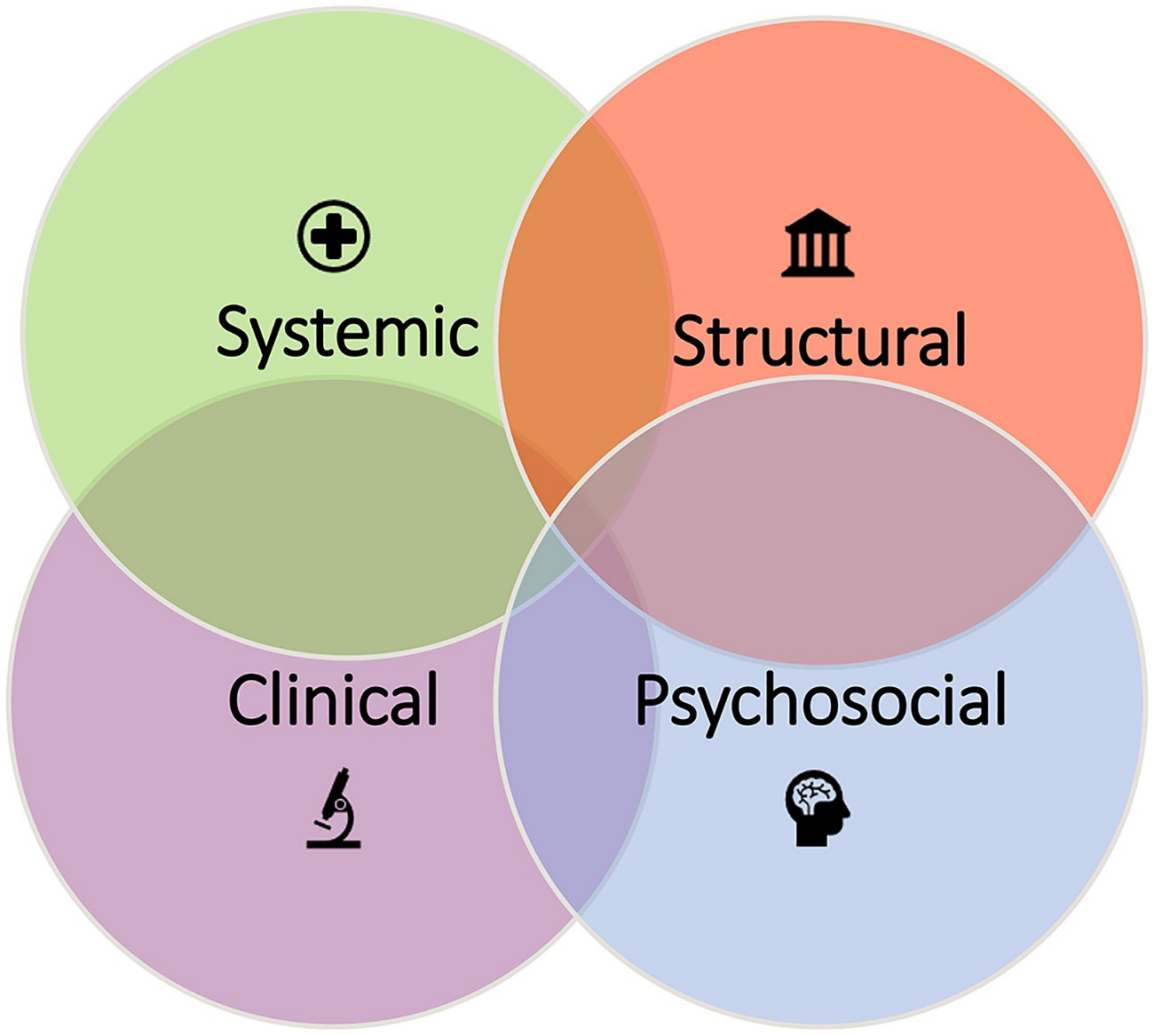
Venn diagram of dimensions of barriers to accessibility of diagnosis and treatment for CD in the US. CD, Chagas disease.

**Fig 3:**
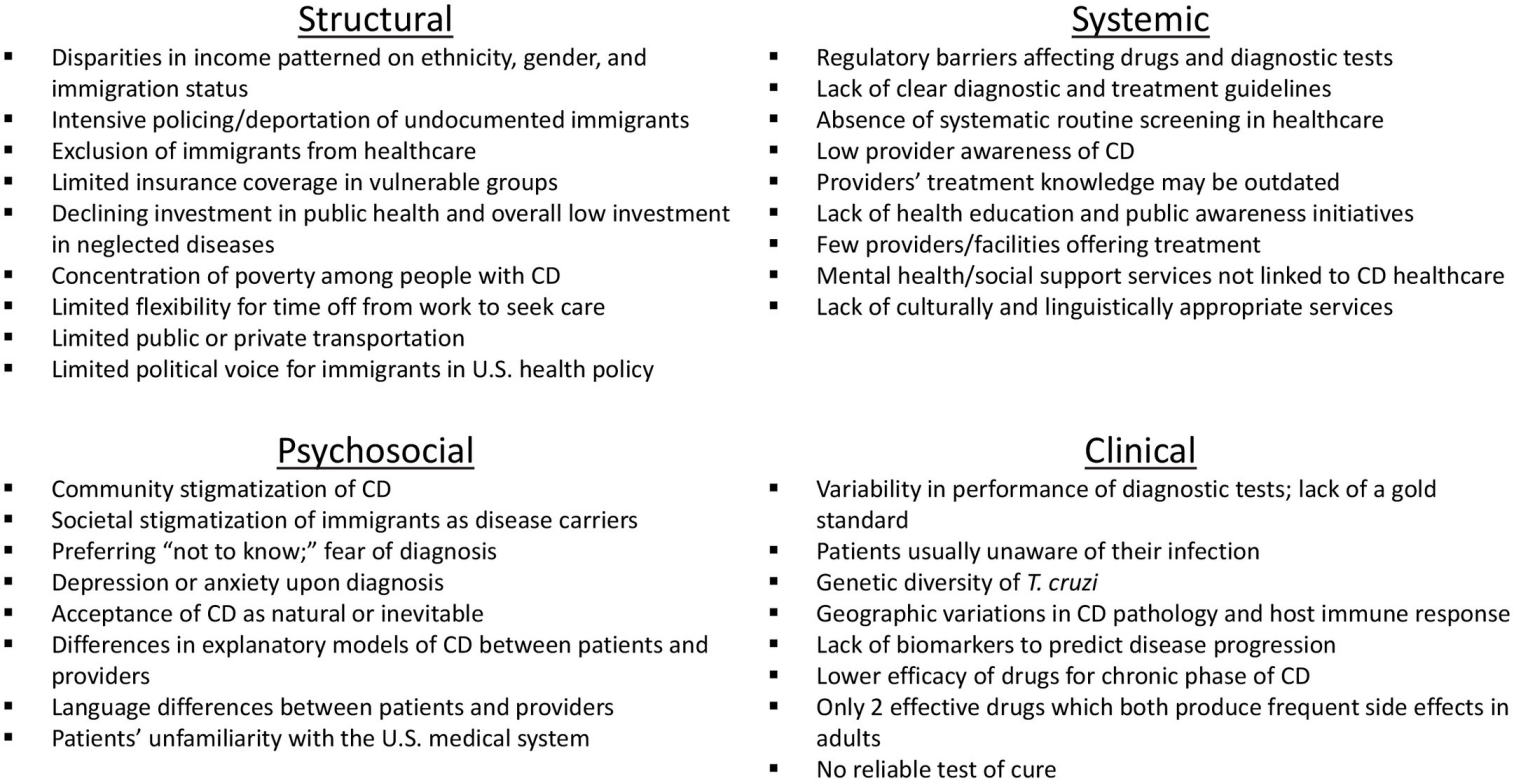
Principal barriers to accessibility of CD diagnosis and treatment in the US by dimension. CD, Chagas disease.

Drawing on both the CECD’s perspective and our analysis of the literature, we identified 34 barriers, which we grouped into 4 overlapping dimensions: clinical, structural, systemic, and psychosocial (Figs 2 and 3). We created this multidimensional framework based on prior work emphasizing the multidimensional aspects of CD [39–42] as a heuristic tool for better comprehending the diverse access barriers relevant to CD in order to develop a more comprehensive, strategic public health response. The dimensions were chosen by the authors in an effort to represent a broad range of perspectives, including medical anthropology, public health, and clinical research. We encourage others to propose alternatives and improve upon our interpretation. Although our model is intended for analysis of CD, it may have applicability to access dynamics for other diseases, particularly those disproportionately affecting marginalized populations.

A framework by Frost and Reich proposes that access to new healthcare technologies for marginalized populations hinges on availability, affordability, adoption, and architecture [43]. These ideas are largely (but not exclusively) reflected in our systemic dimension, although, like Frost and Reich, we consider access as inherently connected to sociopolitical factors. We wanted to expand on some of these issues in greater detail for the case of CD in the US and also incorporate other theoretical approaches. In particular, we wanted to account for the powerful political and economic forces that constrain access to healthcare for vulnerable populations [44], such as Latin American immigrants in the US. We have labeled these structural barriers, drawing from the concept of structural violence, that Farmer and others use to describe the way in which social structures increase risks and limit access to resources for marginalized groups [44–46]. The psychosocial dimension includes cultural and emotional challenges that impact CD patients as they attempt to obtain healthcare and manage the disease. The clinical dimension involves challenging aspects of the disease itself and limitations in current diagnostic and therapeutic tools. Importantly, we propose that these barrier dimensions interact in a synergistic, interdependent manner, as depicted in Fig 2, which is key to understanding the persistent neglect of CD. We provide a preliminary discussion of each dimension next but, again, reiterate that we do not assess the relative weight or importance of each barrier, which could vary considerably not only between groups but individuals.

## Structural barriers

Structural barriers stem from inequalities within the political economic system that restrict access to healthcare for marginalized groups. These barriers are magnified or mitigated according to gender, race, ethnicity, nationality, sexual orientation, language, class, and other social factors [45]. According to Farmer, the disproportionate burden of disease in vulnerable populations, and the limitations placed on their access to healthcare as a result of political and economic forces are aspects of structural violence [44]. Structural barriers can impact the ability of people with CD to obtain health insurance, access and pay for medical services, receive time off, and go to and from medical appointments. This section includes both a general discussion of current healthcare access challenges for Latinos and migrants in the US and specific examples, drawing from the literature and the CECD’s experience, of how structural barriers have affected access for people with CD.

Some Latinos living in the US may have a higher risk for CD because, owing to environmental, geographical, and historical factors, much of Latin America is endemic for CD. US Latinos face various politically and economically rooted impediments to accessing healthcare. Latinos have disproportionately low access to health insurance, both because of restrictions on eligibility for publicly supported health insurance on noncitizens and because they are more likely to be employed in jobs that do not offer health insurance benefits [47, 48]. Latinos also leverage fewer economic resources than non-Latino whites, which in turn may reduce their ability to afford medical services (Table 2). Disparities in income are even more pronounced for Latinos who are foreign born [49].

**Table 2:** Socioeconomic indicators among Latinos versus non-Latino whites in the US, 2014.

Indicator	Latinos	Non-Latino Whites
Median annual income^a^	$42,491	$60,256
Proportion of population living in poverty^a^	23.6%	10.1%
Proportion of population without health insurance^b^	19.9%	7.6%

^a^DeNavas-Walt C, Proctor BD. Income and poverty in the United States: 2014. Washington, DC: United States Census Bureau; 2015.

^b^Barnett JC, Vornovitsky MS. Health insurance coverage in the United States: 2015. Washington, DC: United States Census Bureau; 2016.

Preliminary research suggests structural inequalities profoundly affect many Latino patients with CD in the US. Investigators at the CECD in Los Angeles conducted interviews with 50 Latin American–born CD patients to gauge socioeconomic status and barriers to accessing care [36]. Of 41 patients reporting household income, 26 (63.4%) were below the federal poverty line based on household size. Only 12% had private insurance, and many of the rest only had basic, “emergency” coverage. This was a convenience sample that largely reflects the situation of patients at a Los Angeles safety-net hospital; further research is needed in other settings to get a more complete picture of the socioeconomic status of US CD patients.

Poverty or limited income impacted these patients’ ability to obtain healthcare in myriad ways. An immediate challenge was not having funds to cover co-pays, deductibles, or service fees. Many also lacked transportation, making it difficult to reach appointments [36]. Because 60% of patients in the study did not have a high school education, many were obligated to work low-paying jobs that did not provide paid leave for appointments (nationally, among the lowest-paying 10% of jobs, only 39% provide time off [50]). Similarly, a study in Georgia found that Latin American immigrants would only seek medical care for CD as a last resort, because of concerns about medical expenses and missed time from work [51].

Intensive anti-immigrant rhetoric and the policies that accompany it have significant physical and emotional consequences on undocumented immigrants [52, 53]. Additionally, immigration status significantly impacts access to healthcare (Table 3). Of >300,000 Latin American–born individuals with CD in the US, roughly 30% may be undocumented [2]. Utilization of healthcare by undocumented immigrants is significantly lower than that of the general population [54]; bureaucracy, discrimination, and fear of deportation are important barriers [55]. Although the Affordable Care Act (ACA) of 2010 increased insurance coverage among Latinos by 5.3% [56], it excludes the 11 million undocumented immigrants in the US and certain classes of legal immigrants [57]. Even legal immigrants entitled to coverage under the ACA have hesitated to sign up due to fear of exposing undocumented relatives [56]. In 2017, a national survey found the uninsured rate for citizens was 9%, that of authorized immigrants was 17%, and that of undocumented immigrants was 39% [48]. Even the modest gains made under the ACA have been repeatedly jeopardized by threats to repeal the legislation [58].

**Table 3:** Impact of immigration status on health insurance coverage in California, 2009^a^.

Immigration status	Percent uninsured
US-born citizens	**16.0**
Naturalized citizens	**19.1**
Legal immigrants	**34.8**
Undocumented immigrants	**51.2**

^a^Wallace SP, Torres J, Sadegh-Nobari T, Pourat N, Brown RE. Undocumented immigrants and health care reform: UCLA Center for Health Policy Research; 2012.

Under the Trump administration, efforts to exclude immigrants from healthcare have formed part of a broader, overtly anti-immigration policy. A 2017 Executive Order provides a framework for mass deportation of undocumented immigrants, and the administration has threatened reprisals even for legal immigrants who utilize publicly funded healthcare services [59, 60]. In January 2018, temporary protected status for over 200,000 Salvadorans was terminated, and that same year, an official policy of forced separation of children from parents at the border drew national and international condemnation. These developments, along with highly publicized cases of undocumented immigrants being detained by immigration officials at hospitals, have intensified long-existing fears in immigrant communities. Regardless of status, immigrants may feel reluctant to seek testing and treatment for CD or to leave personal identifying information with providers, out of understandable concern about potential repercussions. Another important consequence is that immigrants with CD may find it particularly challenging to organize and advocate for improvements in health policy and investment for CD within a hostile political climate.

Some CECD patients have reported lack of health insurance as a barrier to obtaining treatment for CD [36], even though Los Angeles County has programs that provide basic coverage for individuals who would otherwise be uninsured. Other patients with insurance have reported delays or difficulties in obtaining authorization for initial CD testing. However, confirmatory testing has been provided at no cost by CDC. In addition to insurance coverage, CECD patients indicated improved transportation and financial resources would facilitate obtaining medical care for CD [36].

## Systemic barriers

Systemic barriers are limitations in the healthcare system’s ability to provide adequate, effective care for people with CD. Manne-Goehler and colleagues identified four main barriers to CD diagnosis and treatment in the US health system: limited diagnosis and follow-up, lack of funds for research and education, low physician awareness, and lack of clear financing mechanisms for patient care. Building on their analysis, we identified nine systemic barriers (as depicted in Fig 3). Next, we describe three in more detail: low provider awareness, lack of systematic screening, and challenges in drug availability. Based on the CECD’s experience, we feel these are important barriers to address, although this is not meant to imply that the other barriers listed in Fig 3 are of less significance, as again, the relative impact of each barrier may vary with local and individual contexts.

### Low awareness of CD among providers and the public

Both providers and patients typically have low familiarity with CD [61–63]. In a national online survey of 1,142 physicians, 47% of obstetrician (OB)/gynecologists (GYNs) and 23% of cardiologists had never heard of CD [61]. Of those who had heard of CD, 44% of cardiologists, 47% of primary care physicians, 48% of transplantation specialists, and 68% of OB/GYNs indicated they were “not at all confident” their knowledge of the disease was current [61]. Another nationwide survey conducted by the American College of Obstetricians and Gynecologists found that 77.9% of 421 OB/GYNs never considered CD diagnosis for patients from endemic countries, while only 8.8% were aware of the risk of congenital transmission [63]. Moreover, US pediatricians seldom consider the risk of congenital CD in infants of parents from Latin America [64]. Even clinicians who have heard of CD may not be familiar with current treatment recommendations.

Historically, treatment for indeterminate CD was not recommended because chronic symptoms were believed to stem from an autoimmune response, not from the presence of the parasite [65]. However, by 2000, this view began to shift as evidence accumulated, supporting parasite persistence as the main trigger of chronic CD pathology [66–68]. Since then, observational studies have shown significant reductions in morbidity and mortality in chronically infected patients who received antitrypanosomal drug therapy [18–20], underscoring the importance of early treatment to eliminate the parasite. Nonetheless, many physicians are not aware of this and continue to operate under the outdated assumption that chronic indeterminate CD in adults should not be treated [68].

Public awareness of CD is also low, even among at-risk groups. The CECD surveyed 2,677 Latin American–born community members while conducting screening and outreach in Los Angeles; 86% had never heard of CD [35]. Campaigns to raise public awareness of CD are practically nonexistent, and patients are unlikely to seek testing while asymptomatic and unaware of CD. In another study of 82 Latin American immigrants in Georgia, only one had previously heard of CD [51]. Though representing different gaps in the health system, low provider and patient awareness are mutually reinforcing. Providers may be unprepared to identify patients at risk and counsel them about testing options. Even if patients become aware of CD (usually through blood donation testing), providers’ lack of awareness can represent an important barrier to obtaining care [25, 27, 36]. Moreover, providers who are under pressure to treat patients quickly may not have adequate time to perform necessary background research to develop treatment strategies.

### Limited testing options

The only systematic screening for *T*. *cruzi* in the US is risk-based screening for donated organs and one-time screening of blood donors (since 2007). Most cases of CD in the US are therefore diagnosed through screening of blood donations, when people who test positive receive a phone call or letter urging them to consult their physician. CD patients in Los Angeles report that providers are widely unfamiliar with CD, frustrating their efforts to obtain care or at least information [36, 69]. Patient-provider language differences, which restrict access and adversely impact quality of care for Latin American immigrants [70], become even more problematic in this context. Furthermore, blood donations underrepresent certain socioeconomic and ethnic groups [71] and may not adequately capture much of the population with CD [72].

Screening in primary care is a highly cost-effective option [73, 74] yet does not systematically occur within the US. A key barrier is the lack of available diagnostic tests. Although numerous assays are available globally, only four have FDA clearance for clinical use, and only one is a rapid, point-of-care assay. Moreover, CD is clinically challenging to diagnose (as we describe more fully later in the article), requiring multiple tests, and there is not currently a clear recommendation on which combination of tests US providers should use. This creates confusion for clinicians and patients. Of the four FDA-cleared tests, one is not currently in production, and another does not have a US distributor.

Greater commercial availability of tests is an urgent need, yet manufacturers may not feel incentivized to develop new tests, obtain FDA clearance, and market their products as long as demand for testing remains low in the clinical setting (which requires a different type of FDA clearance process than blood donation screening). This creates a catch-22 in which testing cannot increase without greater availability of assays, which is not apt to happen unless demand for testing increases. CDC has thus far played a critical role by assisting with confirmatory testing, but if this changes in the future, it is unclear what alternatives would be available through commercial laboratories for diagnostic confirmation.

### Access to antitrypanosmal drugs: Remaining challenges

Only two drugs, both developed nearly 50 years ago, are available for antiparasitic treatment of CD: benznidazole and nifurtimox. Until recently, benznidazole was only available through the CDC via the IND protocol, necessitating considerable paperwork from providers. In August 2017, the FDA granted accelerated approval of benznidazole, a first step towards removing this obstacle [75]. A priority review voucher (PRV, an incentive mechanism for certain neglected diseases, allowing a company to fast-track any other product in its portfolio through the FDA regulatory process) was awarded for the registration of benznidazole to Chemo Group (now InSud Pharma). As part of an agreement between InSud Pharma and the Drugs for Neglected Diseases *initiative*, which supported the application, part of the funds from the sale of the PRV were pledged toward improving access to treatment for CD [76]. Previously, CDC provided benznidazole free of charge; the new distributor, Exeltis (part of InSud Pharma), provides a patient assistance program, which covers the cost of the drug for qualifying uninsured or underinsured patients, and a co-pay assistance program for other patients whose insurance does not cover the cost of the drug, ensuring no patient pays more than $60 out of pocket for a course of treatment (https://www.benznidazoletablets.com/en/).

Nonetheless, key challenges remain, chiefly because the accelerated approval was only for use in children ages 2–12 years old (based on clinical trial data, which more conclusively showed treatment benefit in children). However, over 99% of US patients requesting benznidazole prior to the approval from October 2011 to May 2018 were older than 12 years [28]. Providers can still prescribe benznidazole off-label for patients in other age categories. Meanwhile, the other drug, nifurtimox, is still only available through the CDC-sponsored IND. Because CD is so rarely treated, insurers may not have heard of it and may require special approval before agreeing to cover treatment, adding another layer of obstacles and delays. It may be especially difficult for marginalized, non-English-speaking patients to self-advocate with insurance companies to secure approval for CD treatment. Furthermore, providers have often had to invest considerable time into arranging financing for uninsured patients or securing institutional and payer approval for treatment [27], the costs of which go far beyond the drugs and include laboratory testing (e.g., tests of renal and hepatic function necessitated by the risk of side effects) and posttreatment monitoring.

Clinical management of CD necessitates long-term follow-up to monitor for signs of disease progression. This becomes challenging if patients change providers or, due to socioeconomic constraints, only seek care on an emergency basis. Migrant workers, a particularly high-risk group for CD [77], may find it difficult to maintain follow-up visits with a single provider in a fixed location.

## Clinical barriers

Clinical barriers are biological characteristics of *T*. *cruzi* and the pathophysiology of CD which make the disease particularly challenging to test and treat, as well as limitations of current therapeutic and diagnostic tools. This section details the primary clinical challenges involved in testing and treatment of CD from the perspective of the CECD, while referring to the current medical literature.

### Diagnostic challenges

Because the acute phase is often unrecognized or confused with common viral illnesses, and the indeterminate phase is asymptomatic, people are usually unaware they are infected with *T*. *cruzi*. Patients typically notice symptoms only when their CD is advanced and has begun to impact the heart or other organs, at which point treatment options are more limited. Treatment of patients in the indeterminate phase or with only mild progression has been shown to reduce morbidity and mortality [18–20]. However, in a large clinical trial of older patients with moderate to severe CD cardiomyopathy, etiological treatment with benznidazole was not significantly more effective than placebo [23]. In the absence of routine screening, the disease is not detected in time, and the window of opportunity to provide early treatment that can reduce morbimortality from CD is missed.

Clinical diagnosis of chronic *T*. *cruzi* infection is challenging and relies on detection of antibodies to the parasite [78]. Because no clinically available test has sufficient sensitivity and specificity for single use, WHO recommends diagnosing CD using two different types of immunoassays [79]. Several assays are available on the market, but their performance characteristics vary. Furthermore, the same test may have dramatically different accuracy when used in different populations. This may reflect the genetic diversity of the parasite or geographically driven differences in patients’ immune responses [80, 81].

Most available tests were developed in South American populations, yet the US population with CD represents diverse geographic origins. In a prevalence study in Los Angeles, most people positive for *T*. *cruzi* infection were of Mexican or Central American origin [82]. There is still insufficient information on what combination of commercially available tests will provide sufficient accuracy when used across the range of clinical populations in the US.

### Difficulties in monitoring treatment

Another key limitation involves tools for assessing treatment effectiveness. The time until negative serology following treatment depends on the length of the infection, the patient’s age, and the type of test used. In acute or congenital cases and chronically infected children, seroconversion may be seen within weeks or months, but in adults, it may not occur for over a decade [19]. This makes it challenging to judge the efficacy of drugs or to know in a timely manner which patients will need additional interventions. PCR has been used in clinical trials to measure parasite clearance, whereas parasite persistence provides good evidence of treatment failure. However, it is unclear whether parasite clearance measured by PCR equates to treatment success (PCR used in clinical trials tests patient blood samples for parasite DNA, whereas in chronic CD, the parasite is typically found in tissue), and in any case, this method is challenging to translate into clinical use [83]. Similarly, there is insufficient understanding of which biomarkers can accurately predict CD progression or measure treatment success [84–86]. Although 30%–40% of people infected with *T*. *cruzi* will develop complications from chronic CD, it is unclear which patients are most at risk.

### Tolerability of CD medications

Antiparasitic treatment of CD involves a 60-day regimen of benznidazole or 60–90-day regimen of nifurtimox. Both drugs produce side effects that become more frequent and severe as patient age increases [87]. Benznidazole is usually better tolerated, but it can still lead to severe reactions [13, 88]. The most common side effects from benznidazole are dermatological, although the gastrointestinal and nervous systems can also be affected [89, 90]. Gastrointestinal disorders, including anorexia, and psychiatric and neurological effects, particularly amnesia, are frequently associated with nifurtimox [91]. Adverse effects pose a significant barrier as around 20% of patients are obliged to discontinue treatment, while still others may hesitate to initiate treatment. Physicians could also be reluctant to prescribe antitrypanosmal drugs due to concern over side effects [92].

The CECD has studied adverse events associated with both medications in small cohorts of US adult patients and found, similar to investigations in other settings, that 20%–30% of patients were unable to tolerate treatment. Among 30 patients treated with benznidazole, 16 (53%) experienced rash, with 8 severe cases. Nine (30%) were unable to complete treatment; in 6 cases (20%), this was due to severe reactions [37]. In another study, all 53 patients treated with nifurtimox experienced adverse effects, with a mean of 8.2 per patient, but >90% of these reactions were mild. Eleven patients (20.8%) could not complete treatment; severity and frequency of side effects were both predictors of discontinuation [38]. The possibility of side effects means patients need frequent monitoring and may occasionally require interventions to manage severe reactions.

Research for safer, more effective treatment is an urgent need, yet private sector investment in research and development for CD has historically been negligible [93]. Product development partnerships, collaborations between nonprofit organizations, academic researchers, and other stakeholders, have driven most CD research and development since 2000 [93]. Two promising new medications, posaconazole and fosravuconazole (E1224), were unsuccessful in clinical trials [94, 95], but trial results did provide evidence supporting the efficacy of benznidazole. A recently concluded Phase II trial suggested a course of benznidazole shortened to 2 weeks was as effective as the current 60-day regimen, with significant reduction in side effects, but this needs to be confirmed in a larger study [96, 97]. Meanwhile, new leads are being developed through innovative public–private partnerships, including one effort that has used artificial intelligence to screen millions of compounds [98]. Another clinical study is evaluating fexinidazole (https://www.dndi.org/diseases-projects/portfolio/fexinidazole-chagas/). Ultimately, a safe, effective, easily administered treatment will be instrumental to eliminating access barriers for CD patients.

## Psychosocial barriers

This section assesses psychosocial challenges related to CD; because US research on this topic is still limited, studies from other countries are referenced where pertinent. Beyond its biological impact, CD affects the social life and emotional health of people with the infection, as Oliveira eloquently describes [41]. Neglected tropical diseases are highly stigmatized in many settings and cultural contexts [99]. Studies in Latin America highlight stigmatization of people with CD, particularly in urban areas, because of the disease’s association with rurality and poverty [100–102]. CD diagnosis has even impacted patients’ employment in some settings [42, 103, 104]. In other cases, stigma associated with CD has contributed to avoidance of testing [40, 101].

In the US, stigmatization of CD is interwoven with discrimination and negative societal perceptions of immigrants. Although CD has been present in the southern US for centuries [105] as a natural zoonosis, a common misconception of CD is that it is a foreign, imported disease. The association of Latin American immigrants with CD has contributed to negative stereotypes of this population, which already suffers from intense political exclusion and marginalization. In 2015, then-candidate Donald Trump claimed “tremendous infectious disease is pouring across the border” [106], while anti-immigrant news outlets blame immigrants for bringing CD and other diseases into the US.

Stigmatization and understandable fears of a life-threatening disease create a considerable emotional burden for people diagnosed with CD [100, 107]. US patients describe feelings of isolation, anguish, and abandonment because of their diagnosis [69, 108]. Consequently, patients may feel it is preferable not to know if they have CD [51]. Many battle depression and anxiety over their diagnosis [108]. These struggles are exacerbated when patients feel isolated from family, which Latin American–born patients at the CECD describe as a key challenge in adjusting to life in the US. Indeed, family members play a crucial role in helping patients obtain healthcare, by providing transportation, translation, and care for patients weakened due to side effects from treatment or the impacts of advanced CD [36].

Linguistic and cultural differences between patients and providers are another key challenge. In a CECD study, a majority of CD patients identified language as the most difficult aspect of adjusting to life in the US [36]. Providers’ linguistic capabilities may not be adequate for navigating a discussion about CD with patients who are not fully fluent in English, particularly if provider awareness is low. The story of a woman who attempted to request CD testing and was tested for Lyme disease instead is illustrative of the communication problems that can arise between doctors and patients, regardless of language fluency [36].

Moreover, patients may have perspectives on health and disease that differ considerably from the biomedical model in which US clinicians are trained. Minneman and colleagues identified three potential phases of healthcare-seeking behaviors for CD in a sample of Latin American immigrants: (1) utilizing traditional remedies, (2) waiting, and if symptoms failed to improve, (3) seeking care from an alternative or mainstream healthcare provider [51]. Among 50 patients interviewed at the CECD, use of traditional remedies for other, better-known illnesses was common but was rarely reported for CD [108]. This could be due to low awareness of the disease among Latin Americans living in Los Angeles. In contrast, a study in Bolivia, where familiarity with the disease is higher, identified 33 traditional or alternative remedies for CD [109].

## Discussion

Since Carlos Chagas first described CD in 1909, the disease’s relationship with the social sphere has been described. Other researchers have pointed out that while CD has biomedical, epidemiological, sociocultural, and political dimensions, providers focus almost exclusively on its biological aspects [39, 40]. However, research in Latin America and Europe has begun to explore both the important emotional impact of CD and the relationship of social, cultural, political, and economic factors to treatment access [40, 101, 107, 110]. A study of mostly undocumented Bolivian CD patients in Switzerland detailed high levels of depression and anxiety; 89.1% lived below the Swiss poverty line and 72.3% lacked health insurance [111]. Another Argentinian study suggests that patients with less education and insurance coverage had worse clinical outcomes following treatment [112].

As the following examples illustrate, multifactorial barriers to diagnosis and treatment for CD in the US must also be understood as interdependent and synergistic. For instance, depression and anxiety may be caused by CD diagnosis (psychosocial barrier), but they are also potential side effects of treatment with nifurtimox (clinical barrier), and few programs in the US or elsewhere address the emotional impact of the disease (systemic barrier). A patient in a job without benefits (structural barrier) cannot take time off from work if weakened by side effects during treatment (clinical barrier). In other words, and exemplifying the synergistic interaction of barriers, dealing with side effects is primarily a clinical challenge for a wealthy individual but could be an economic (as well as clinical) challenge for a low-income person. Latin American–born community members may be fearful of getting screened due to fear about reprisals from the government (structural barrier) and may prefer not to know if they have a deadly disease (psychosocial barrier) for which treatment is not always effective (clinical barrier). Few assays are available in the US for clinicians’ use (systemic barrier), and tests are often developed using parasite strains that are not common in US patients (clinical barrier). On a societal level, stigmatization of CD as a disease of immigrants reinforces structural barriers that limit healthcare access for this population and helps perpetuate the lack of a public health response to the disease.

**Fig 4:**
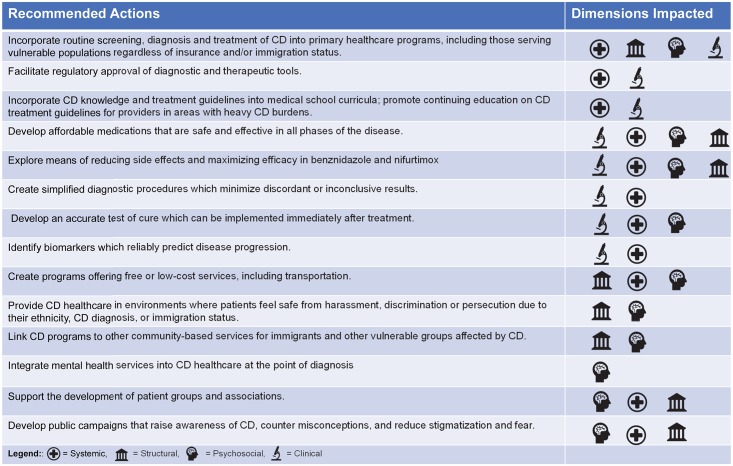
Recommendations for a comprehensive approach to improving access to care for CD in the US. The first column proposes actions, and the second column indicates barrier dimensions most impacted by each action. In the second column, the icons each represent a barrier dimension (see Fig 2). The first icon listed is the dimension primarily targeted by the action. For example, the first action, “Incorporate routine screening, diagnosis and treatment of CD into primary healthcare programs, including those serving vulnerable populations regardless of insurance and/or immigration status,” focuses on the systemic dimension yet ameliorates structural barriers (by providing services more easily accessed by vulnerable communities), psychosocial barriers (by integrating services at the community level, potentially mitigating stigma and fear), and can even improve the clinical dimension by assuring a larger patient population is available for involvement in clinical studies. CD, Chagas disease.

Because of these interrelationships, there are no quick fixes, and focusing solely on one dimension may not be sufficient to control CD as a public health concern. Multidimensional barriers continually constrain the CECD’s ability to provide testing and treatment, despite intense dedication from its staff and volunteers. In the CECD’s experience, structural barriers prevent patients from coming to appointments or paying for testing, systemic barriers lead to uncertainty about how to obtain testing for patients, clinical barriers make it difficult to treat patients and tell them if they have been cured, and psychosocial barriers create anxiety for patients. Expanding CD treatment in the US will require involvement from a broad range of stakeholders, including preclinical and clinical researchers, social scientists, healthcare policy experts, healthcare providers, immigrant and patient rights advocates, government, and industry. Fig 4 proposes several potential actions at national, state, and local (provider) levels that begin to address the multidimensional barriers we have described. Some structural and psychosocial barriers, such as income disparities or societal stigmatization of immigrants as disease carriers, will not be fully resolved without profound social and cultural change. We simply propose that programs show sensitivity to these dimensions and design actions that help patients navigate through such barriers so they can access care. For example, supporting low-cost testing and medication helps low-income patients, while ensuring accurate, reliable information about CD is available to the public could help reduce stigma.

Nationally, several recommendations are proposed by Manne-Goehler and colleagues, including development of a US Chagas Task Force that, working in tandem with CDC, would develop a straightforward screening guideline, promote and coordinate research and surveillance, and carry out a provider education campaign [27]. An important need is funding for continued research on the epidemiology of CD in the US and on improved diagnostic tools, especially point-of-care tests, which can accurately assess *T*. *cruzi* infection in the US’s heterogeneous patient population. Testing and treatment processes have historically been centralized through CDC. However, this situation changed with the commercial launch of benznidazole in May 2018; providers now may order benznidazole directly from the distributor, Exeltis. While CDC continues to provide assistance to providers with testing and other consultations, increasing the level of screening and testing to a level at which it could cover the entire population at risk would represent a potentially very large volume for a single government agency. Creation of a network of referral centers in high-burden states (California, Texas, Florida, New York, and Virginia/DC), linked to primary healthcare providers accessible to patients with CD, would be one way to scale up testing and treatment. CDC could continue to provide leadership at the national level. These state-level reference centers may be better positioned to provide health education, provider training, and diagnostic and clinical consultation that are tailored to the specific needs of each state’s population. This is important because patient populations, insurance coverage, and even potential transmission routes vary considerably between states.

At a local level, there are also steps programs can take to address different barrier dimensions and increase patient access. In Los Angeles, the CECD has provided screening through outreach activities in close partnership with churches, which provide a safe and trusted location for members of the Los Angeles Latin American community [34]. The Strong Hearts project in Boston conducts outreach through churches and has integrated CD screening into primary healthcare. Certain aspects of successful international models could also be adapted to the US context. For example, the Chagas Disease and Heart Failure Outpatient Clinic at the Federal University of Pernambuco Hospital in Recife, Brazil, has adopted a comprehensive treatment model with a multidisciplinary team that includes psychologists and social workers; the clinic works closely with a local CD patient association and provides cost-effective care while achieving high patient adherence [41]. In the US, a multidisciplinary approach will also be key to addressing multidimensional barriers. We suggest a model of care in which primary healthcare personnel manage screening, diagnosis, and etiological treatment, as recommended by WHO [113]. Pediatricians and OB/GYNs detect, monitor, and manage congenital CD, whereas cardiologists and other specialists treat complications from advanced CD. Mental health specialists and social workers manage psychosocial impacts, and health educators work to raise awareness of CD, in both instances, collaborating closely with patient groups.

Our review of barriers literature has significant limitations and should be viewed as an exploratory assessment subject to interpretation bias. Our ability to gauge the extent of research, particularly on clinical barriers in the US, was likely limited by our search terms, with the end result that most of the sources in Table 1 discuss systemic barriers. Still, the limited number of sources we found suggests a need for more research on structural and psychosocial dimensions of CD in the US. Although we attempted to use a systematic approach to describe access-related literature on US CD, our desire to provide in-depth context for the different dimensions of barriers necessitated drawing on a much wider range of information sources than was encompassed in the literature search. Finally, the CECD’s experience is largely reflective of its Los Angeles setting; local dynamics could differ considerably in other parts of the US.

Because CD is one of many issues afflicting marginalized Latin American–born residents of the US, including poverty, disenfranchisement, and discrimination, efforts to eliminate the disease as a public health problem must align with broader social and political movements that affirm healthcare as a human right while addressing root-cause socioeconomic disparities that limit access to treatment. However, because CD is also part of the natural environment in the US, more research is needed to understand the impact of autochthonous transmission. US-born patients who acquire the disease from kissing bugs in the US face many of the barriers described here when they attempt to obtain healthcare. Characterization of CD as a “disease of immigrants” not only risks perpetuating negative stereotypes of immigrants in an increasingly anti-immigrant political culture but also reinforces the neglect of the nonimmigrant sector of the US CD population.

The current paradigm, in which only a tiny fraction of CD cases is detected and treated, leads to a heavy yet avoidable burden in morbidity and mortality. This situation will not change without wide scale-up of more integrated strategies, which in turn hinges on commitment from government and public health systems, increased scientific research for improved treatment and diagnostic tools, greater accessibility of medications, broad awareness campaigns targeting both patients and providers, and a comprehensive treatment strategy that addresses the biological, psychological, and social impacts of the disease.

## Supporting information

S1 Data US Chagas barriers literature search results. (XLSX)

S1 Table Quick reference of selected sources used for information on barriers. (DOCX)

**Key Learning Points**

- Less than 1% of people with CD in the US are diagnosed and treated; the reasons for this are diverse, complex, and intertwined. We identified 34 barriers, which we divided into four main dimensions, explained below. To scale up access to treatment for CD, all four barrier dimensions should be addressed.
- **Structural barriers** are rooted in widening political and economic inequalities that increasingly limit the ability of immigrants and other vulnerable groups to afford or access proper healthcare.
- **Systemic barriers** are gaps in the health system, including the lack of systematic screening in healthcare facilities, the limited availability of diagnostics, and very low awareness of CD among healthcare professionals.
- Key **clinical barriers** are limitations in the safety and efficacy of antitrypanosomal drugs for chronically infected adults and the lack of a reliable test of cure.
- **Psychosocial barriers** include stigmatization of people with CD (often in tandem with anti-immigrant discrimination) and fears and anxieties about the disease, which might discourage affected people from seeking treatment.

**Top Five Papers**

1. Manne-Goehler J, Reich MR, Wirtz VJ. Access to Care for Chagas Disease in the United States: A Health Systems Analysis. Am J Trop Med Hyg. 2015;93:5.
2. Montgomery SP, Starr MC, Cantey PT, Edwards MS, Meymandi SK. Neglected Parasitic Infections in the United States: Chagas Disease. Am J Trop Med Hyg. 2014;90(5):814–8.
3. Sanchez DR, Traina MI, Hernandez S, Smer AM, Khamag H, Meymandi SK. Chagas Disease Awareness among Latin American Immigrants Living in Los Angeles, California. Am J Trop Med Hyg. 2014;91(5):915–9.
4. Stimpert KK, Montgomery SP. Physician Awareness of Chagas Disease, USA. Emerg Infect Dis. 2010;16(5):871–2.
5. Forsyth CJ, Hernandez S, Flores CA, Roman MF, Nieto JM, Marquez G, et al. "It’s Like a Phantom Disease": Patient Perspectives on Access to Treatment for Chagas Disease in the United States. Am J Trop Med Hyg. 2018;98(3):735–41.
