# Supplementary material for: Proposed multidimensional framework for understanding Chagas disease healthcare barriers in the United States
Source: PLoS Negl Trop Dis. 2019 Sep 26;13(9):e0007447. doi: 10.1371/journal.pntd.0007447 (PMC6762052; doi:10.1371/journal.pntd.0007447)
Supplement: S1 Table — (DOCX) [file pntd.0007447.s002.docx]

**Supplemental Table: Quick Reference of Selected Sources Used for Information on Barriers***

| **Barrier** | **Selected Sources** |
| --- | --- |
| **Structural Barriers** | |
| Disparities in income patterned on ethnicity, gender, and immigration status | https://www.pewresearch.org/fact-tank/2016/07/01/racial-gender-wage-gaps-persist-in-u-s-despite-some-progress/ |
| Intensive policing/deportation of undocumented immigrants | Holmes 2013; DeLeon 2015 |
| Exclusion of immigrants from healthcare | Chavez 2012 |
| Limited insurance coverage in vulnerable groups | Artiga et al 2019; Hacker et al 2015; Wallace et al 2012 |
| Declining investment in public health and overall low investment in neglected diseases | Trouiller et al 2002 |
| Concentration of poverty among people with CD | Forsyth et al. 2018; Jackson et al. 2012 |
| Limited flexibility for time off from work to seek care | CECD experience; Forsyth et al. 2018 |
| Limited public or private transportation | CECD experience; Forsyth et al. 2018 |
| Limited political voice for people with NTDs | WHO 2010 |
| **Psychosocial Barriers** | |
| Community stigmatization of CD | CECD experience; Ventura-Garcia et al. 2013 |
| Societal stigmatization of immigrants as disease carriers | Ventura-Garcia et al. 2013 |
| Preferring “not to know;” fear of diagnosis | Minneman et al. 2013 |
| Depression or anxiety upon diagnosis | Jackson et al. 2012; Forsyth et al. 2018 |
| Acceptance of CD as natural or inevitable | Sanmartino 2009; Forsyth 2017 |
| Differences in explanatory models of CD between patients and providers | Forsyth 2015 |
| Language differences between patients and providers | CECD experience; Forsyth et al. 2018 |
| Patients’ unfamiliarity with the U.S. medical system | CECD experience |
| **Systemic Barriers** | |
| Regulatory barriers affecting drugs and diagnostic tests | Manne-Goehler et al. 2015 |
| Lack of health education and public awareness initiatives | Sanchez et al. 2014 |
| Lack of clear diagnostic and treatment guidelines | CECD experience; Manne-Goehler et al. 2015 |
| Absence of systematic routine screening in healthcare | Manne-Goehler et al. 2015 |
| Low provider awareness of CD | Stimpert et al. 2010; Verani et al. 2010; Amstutz et al. 2017; Edwards et al. 2018 |
| Providers’ treatment knowledge may be outdated | CECD experience; Viotti et al. 2014 |
| Few providers/facilities offering treatment | Manne-Goehler et al. 2015 |
| Mental health/social support services not linked to CD healthcare | CECD experience |
| Lack of culturally and linguistically appropriate services | CECD experience |
| **Clinical Barriers** | |
| Variability in performance of diagnostic tests; lack of a gold standard | CECD experience |
| Patients usually unaware of their infection | <https://www.cdc.gov/parasites/chagas/disease.html> |
| Genetic diversity of T. cruzi | Messenger et al. 2015 |
| Geographic variations in CD pathology and host immune response | Verani et al. 2009; Messenger et al. 2015 |
| Lack of biomarkers to predict disease progression | Requena-Mendez et al. 2013; Chatelain 2016 |
| Lower efficacy of drugs for chronic phase of CD | Bermudez et al. 2015 |
| Only 2 effective drugs which both produce frequent side effects in adults | Miller et al. 2015; Forsyth et al. 2016 |
| No reliable test of cure | Pinazo et al. 2015 |

* Only key sources are listed to orient the reader; for more complete information see the article references.
